# East Africa International Center of Excellence for Malaria Research: Summary of Key Research Findings

**DOI:** 10.4269/ajtmh.21-1285

**Published:** 2022-10-13

**Authors:** Joaniter I. Nankabirwa, John Rek, Emmanuel Arinaitwe, Jane Frances Namuganga, Sam L. Nsobya, Victor Asua, Henry D. Mawejje, Adrienne Epstein, Bryan Greenhouse, Isabel Rodriguez-Barraquer, Jessica Briggs, Paul J. Krezanoski, Philip J. Rosenthal, Melissa Conrad, David Smith, Sarah G. Staedke, Chris Drakeley, Teun Bousema, Chiara Andolina, Martin J. Donnelly, Moses R. Kamya, Grant Dorsey

**Affiliations:** ^1^ Infectious Diseases Research Collaboration, Kampala, Uganda;; ^2^ Department of Medicine, Makerere University, College of Health Sciences, Kampala, Uganda;; ^3^ Department of Epidemiology and Biostatistics, University of California San Francisco, San Francisco, California;; ^4^ Department of Medicine, University of California San Francisco, San Francisco, California;; ^5^ Institute for Health Metrics & Evaluation, University of Washington, Seattle, Washington;; ^6^ Department of Clinical Research, London School of Hygiene & Tropical Medicine, London, United Kingdom;; ^7^ Department of Medical Microbiology, Radboud University Nijmegen Medical Centre, Nijmegen, The Netherlands;; ^8^ Department of Vector Biology, Liverpool School of Tropical Medicine, Liverpool, United Kingdom

## Abstract

The Program for Resistance, Immunology, Surveillance, and Modeling of Malaria (PRISM) has been conducting malaria research in Uganda since 2010 to improve the understanding of the disease and measure the impact of population-level control interventions in the country. Here, we will summarize key research findings from a series of studies addressing routine health facility-based surveillance, comprehensive cohort studies, studies of the molecular epidemiology, and transmission of malaria, evaluation of antimalarial drug efficacy, and resistance across the country, and assessments of insecticide resistance. Among our key findings are the following. First, we found that in historically high transmission areas of Uganda, a combination of universal distribution of long-lasting insecticidal-treated nets (LLINs) and sustained indoor residual spraying (IRS) of insecticides lowered the malaria burden greatly, but marked resurgences occurred if IRS was discontinued. Second, submicroscopic infections are common and key drivers of malaria transmission, especially in school-age children (5–15 years). Third, markers of drug resistance have changed over time, with new concerning emergence of markers predicting resistance to artemisinin antimalarials. Fourth, insecticide resistance monitoring has demonstrated high levels of resistance to pyrethroids, appreciable impact of the synergist piperonyl butoxide to pyrethroid susceptibility, emerging resistance to carbamates, and complete susceptibility of malaria vectors to organophosphates, which could have important implications for vector control interventions. Overall, PRISM has yielded a wealth of information informing researchers and policy-makers on the malaria burden and opportunities for improved malaria control and eventual elimination in Uganda. Continued studies concerning all the types of surveillance discussed above are ongoing.

## INTRODUCTION

Uganda is emblematic of other high malaria burden countries in Sub-Saharan Africa. Malaria is endemic in over 95% of the country and in the remaining highland areas, transmission is unstable and epidemic-prone.^1^ Malaria is the leading cause of morbidity and mortality in Uganda, accounting for 30–50% of outpatient visits and 15–20% of hospital admissions.^2^ Like many other African countries, malaria control efforts in Uganda have focused on long-lasting insecticide-treated nets (LLINs), indoor residual spraying (IRS) of insecticide, and effective case management with artemisinin-based combination therapies (ACTs). The country has conducted three mass LLIN distribution campaigns (2010–2011, 2013–2014, and 2020–2021) leading to an increase in the proportion of households owning at least one LLIN from 47% in 2009 to 83% in 2018.^1^^,^^3^ Uganda’s IRS program was reinitiated in 2006, after a gap of 40 years, and is currently being implemented in 14 of 112 districts. Uganda adopted artemether-lumefantrine (AL) as its first-line therapy in 2004, and AL is provided free of charge at public health facilities. Despite roll out of effective control intervention, the burden of malaria remains high in Uganda. The country currently ranks third in terms of number of malaria cases and number of malaria deaths globally.^4^

The Program for Resistance, Immunology, Surveillance, and Modeling of Malaria in Uganda (PRISM) was established in 2010 and represents the East African region of the International Center of Excellence for Malaria Research (ICEMR) network with a focus on Uganda. Uganda is emblematic of the challenges faced by high-burden countries, where routine surveillance systems have limited ability to assess trends in the burden of malaria or to monitor the impact of control interventions. Through PRISM, researchers have implemented a comprehensive malaria surveillance program including enhanced health facility-based surveillance and detailed longitudinal cohort studies in areas with differing transmission intensities. Complementary laboratory-based studies have included surveillance for markers of antimalarial drug and insecticide resistance, serologic measures of malaria exposure, highly sensitive molecular assays for the detection of asexual- and sexual-stage parasites, and membrane feeding assays to assess human to mosquito transmission. These studies have greatly improved our understanding of the epidemiology of malaria in Uganda and of the impact of control interventions. In recent years, the program has expanded its malaria surveillance work and the scope of longitudinal studies to address more fundamental questions about interactions among the parasite, mosquito vector, and human host. Here, we report a summary of key research findings from our PRISM projects and some related studies from our group, which together offer a comprehensive understanding of malaria in Uganda, one of the highest malaria burden countries in the world.

### Health facility-based malaria surveillance.

Malaria surveillance, which encompasses monitoring and evaluation of malaria control efforts, is essential to guide program planning and management. Malaria surveillance in Uganda is mainly dependent on passive case detection at health facilities as part of a country’s routine health management information system (HMIS). There are several strengths in conducting surveillance at health facilities: available data provide direct measures of morbidity, are collected continuously over time, and cover a wide geographic area.^5^ However, as is the case in many African countries, HMIS data in Uganda is provided as aggregate numbers from public facilities that are often inadequate for monitoring disease trends. Health management information system data also often has incomplete reporting, poor accuracy, and limited diagnostic testing.

**Figure 1.**
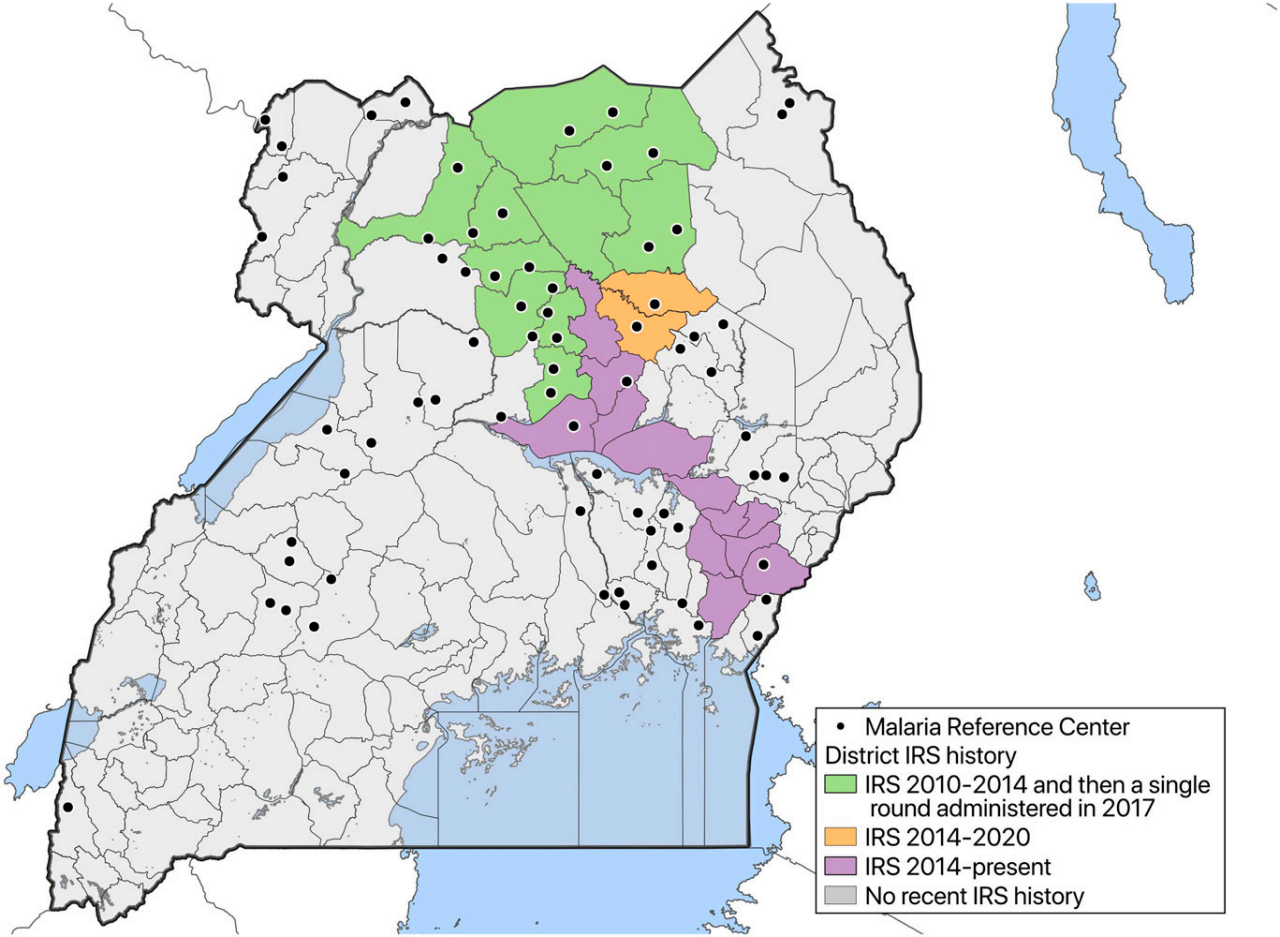
Map of Uganda with locations of sites where health facility-based malaria surveillance is being conducted. This figure appears in color at www.ajtmh.org.

In 2006, the Uganda Malaria Surveillance Program (UMSP) was established to collect high-quality malaria surveillance data at six health facilities, known as Malaria Reference Centers (MRCs), in collaboration with the Uganda National Malaria Control Division (NMCD).^6^ From 2014 to 2020, this surveillance network was gradually expanded to include 70 MRCs located in 38 districts across the country, with support from PRISM (Figure 1). At the participating facilities, individual-level data for all outpatients that present to these MRCs are collected using standardized registers provided by the Ministry of Health and entered on site into an electronic database. These data include patient demographics (age, sex, and village of residence), results of laboratory tests, diagnoses given, and treatments prescribed. Uganda Malaria Surveillance Program conducts site support supervision, mentorship of health workers in malaria diagnosis and laboratory testing, and training to ensure the collection of high-quality malaria surveillance data. This enhanced malaria surveillance using data routinely collected from health facilities provides a powerful tool to monitor trends in malaria burden across time and space.

**Figure 2.**
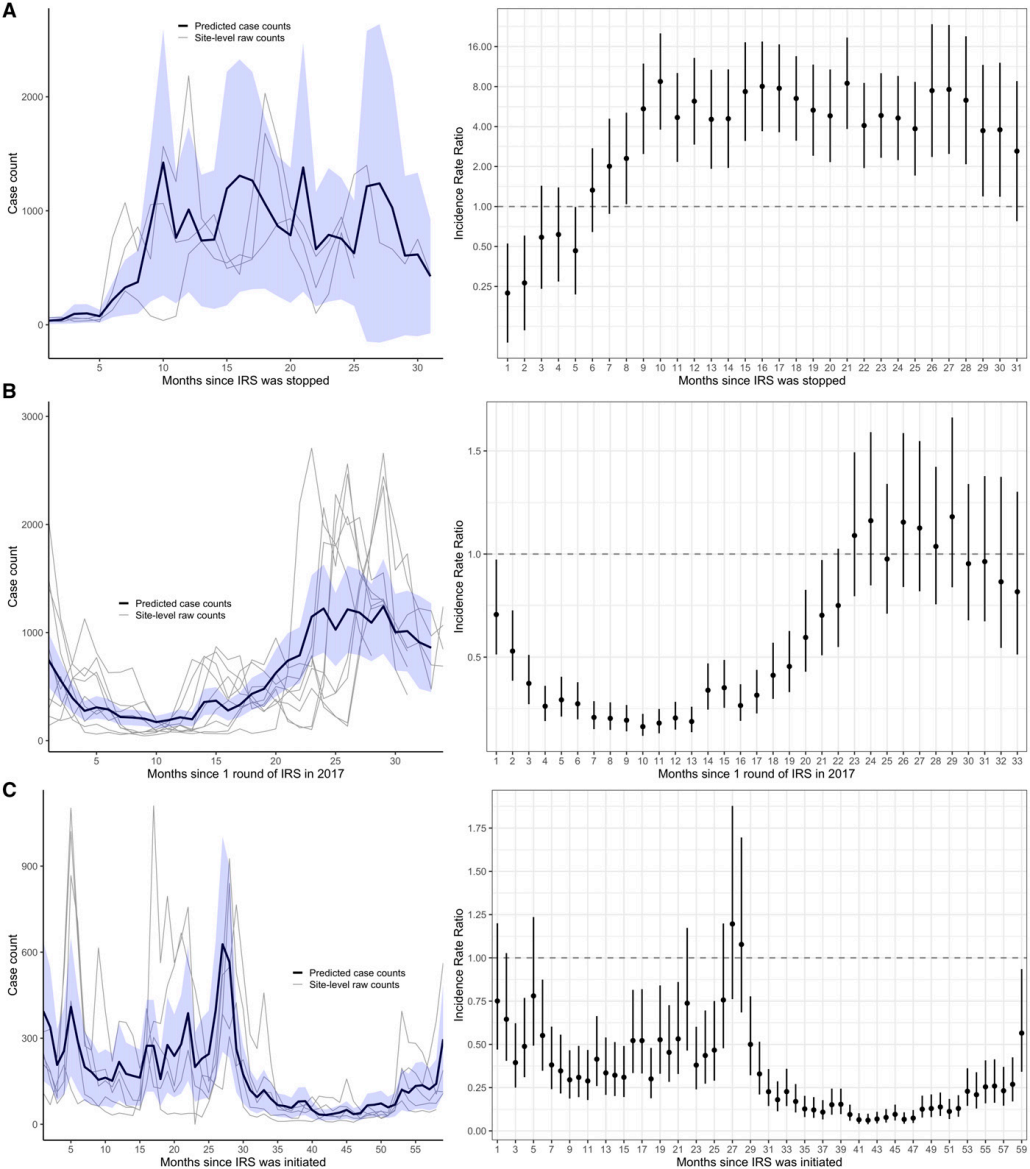
(**A**) Adjusted Incidence rate ratio (IRR) and predicted case counts from multilevel negative binomial model assessing the impact of withdrawing IRS after 5 years of sustained use, (**B**) restarting IRS with a single round, and (**C**) initiating and sustaining IRS. Incidence rate ratios were adjusted for time-varying variables that impact malaria burden and malaria case detection at the health facility including monthly rainfall at the health facility lagged by 1 month, indicator variables for month of the year (to adjust for seasonal effects), the proportion of tests that were RDTs in that month (vs. microscopy), and the number of individuals who attended the health facility but were not suspected of having malaria in that month (to adjust for potential changes in care-seeking behaviors over time). The blue shaded regions represent the 95% confidence interval (CI) around the mean predicted case counts across sites from the adjusted regression model. Gray lines represent observed monthly case counts from individual sites. Vertical bars represent the 95% CI around adjusted IRR. This figure appears in color at www.ajtmh.org.

In 2020, we used data from this enhanced malaria surveillance system to assess the impact of starting and stopping IRS of insecticide on malaria burden in Northern and Eastern Uganda.^7^ Stopping IRS at three sites (Figure 2A) resulted in a 5-fold increase in malaria incidence within 10 months relative to the period before IRS was stopped (adjusted IRRc = c5.24, 95% CI 3.67–7.50); restarting IRS at nine sites (Figure 2B) led to an over 5-fold decrease in malaria incidence within 8 months (adjusted IRRc = c0.17, 95% CI 0.15–0.20). At five sites, where IRS was initiated and sustained over 5 years (Figure 2C), malaria incidence had dropped by 85% in the fourth and fifth years of sustained use relative to the pre-IRS period (adjusted IRRc = c0.15, 95% CI 0.12–0.18).

Since 2018, emphasis has been placed on collecting data on village of residence for all patients presenting to the MRCs. These data—along with the identification of target areas around the MRCs and enumeration surveys to estimate the populations of these target areas—have allowed for the estimation of malaria incidence within these target areas. Currently, data from our enhanced malaria surveillance network are being used as a platform for a cluster randomized trial evaluating the effect of two different types of newer generation LLINs on malaria incidence. A total of 64 clusters (MRC target areas) covering 32 high-burden districts have been randomized in a 1:1 ratio in blocks of two by district to receive one of each LLIN type: Permanet 3.0^®^ LLINs containing deltamethrin and piperonyl butoxide (PBO) or Royal Guard^®^ LLINs containing alpha-cypermethrin and pyriproxyfen. LLINs were distributed during Uganda’s 2020–2021 universal coverage campaign. By using malaria incidence measured within the target areas as the primary outcome for this trial, we will monitor temporal trends in malaria morbidity, and compare absolute disease burden in each trial arm.

In summary, data from our enhanced malaria surveillance provides an efficient means to quantify the impact of malaria control interventions at the population-level using observational study designs (i.e., “before and after” comparisons) or cluster randomized trials through coordination with implementing partners. This approach is more cost-effective and timely compared with the Demographic and Health Surveys that are conducted every 5 years and yet provides more comprehensive information beyond what is provided by the routine HMIS.

### Comprehensive cohort studies.

Comprehensive cohort studies, including clinical and entomological assessments, provide a powerful design for generating longitudinal data on measures of malaria transmission, infection, and disease. We have conducted comprehensive malaria surveillance in cohorts from Tororo District, Uganda, an area with historically high transmission intensity that saw a dramatic decline in the burden of malaria following the implementation of intensive malaria control interventions (EIR = 562 infective bites/person/year in 2006 and 310 infective bites/person/year in 2011).^8^^,^^9^ Prior to 2013, malaria control in Tororo was limited to the distribution of LLINs through antenatal care services, promotion of intermittent preventive treatment during pregnancy, and malaria case management with AL. In November 2013, universal distribution of free LLINs was conducted as part of a national campaign, and a similar campaign was repeated in May 2017. Indoor residual spraying with the carbamate bendiocarb was first initiated in December 2014–January 2015, with additional rounds administered in June–July 2015 and November–December 2015. In June–July 2016, the insecticide delivered through IRS was changed to the organophosphate pirimiphos-methyl (Actellic), which was repeatedly delivered in June–July 2017, June–July 2018, and March–April 2019.

Two consecutive cohort studies (referred to as the “PRISM” cohorts) were conducted in randomly selected households from Nagongera subcounty, Tororo District.^9^^,^^10^ In PRISM 1, all children aged 0.5–10 years of age were enrolled from 100 houses and followed from October 2011 to September 2017. In PRISM 2, all household members were enrolled from 80 houses and followed from October 2017 to October 2019. All cohort members were given access to an LLIN at enrollment and followed for all their healthcare needs in a dedicated study clinic open 7 days/week. Routine visits were conducted every 1–3 months and included the collection of blood for assessment of parasitemia by microscopy and molecular methods (loop-mediated isothermal amplification [LAMP] in PRISM 1 and quantitative PCR [qPCR] in PRISM 2). Malaria was defined as a recent fever (defined as tympanic temperature > 38.0°C or history of fever in the previous 24 hours) and a positive thick blood smear by light microscopy and managed according to national guidelines. The cohorts were dynamic such that all newly eligible members from participating households were enrolled. In all cohort households, mosquitoes were collected using CDC light traps, monthly in PRISM 1 and every 2 weeks in PRISM 2. Entomological assessments included quantifying the number of female *Anopheles* and the detection of sporozoites using ELISA.

**Figure 3.**
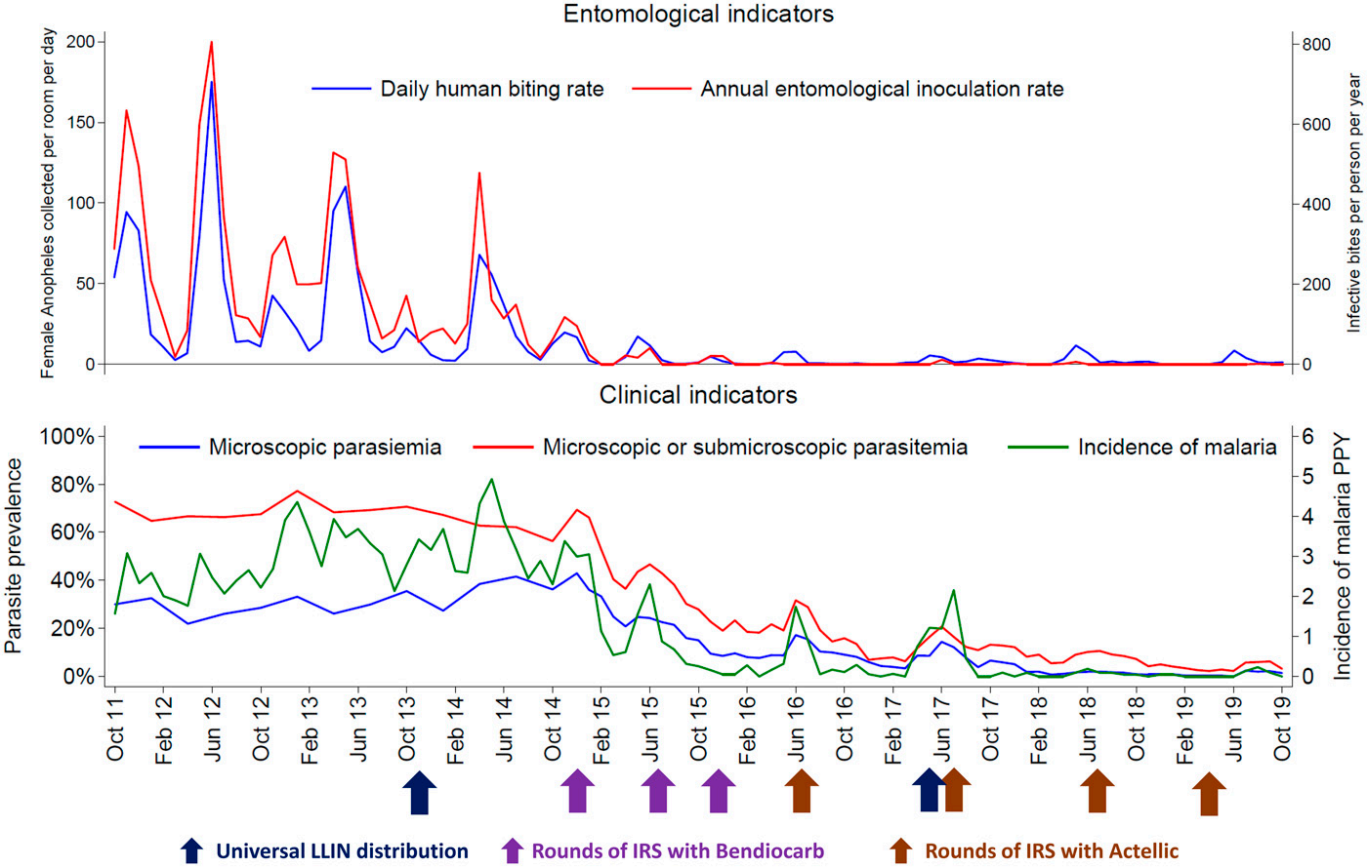
Temporal trends in entomological and clinical indicators. Clinical indicators limited to children 0.5–10 years of age. Measures of parasite prevalence based on active surveillance at the time of all enrollment and routine clinic visits. Incidence of malaria based on passive surveillance. This figure appears in color at www.ajtmh.org.

Following the implementation of IRS, measures of transmission, infection, and disease reduced dramatically (Figure 3). Pre-IRS, the daily anopheline human biting rate (HBR) was 34.3 and the annual entomological inoculation rate (EIR) was 238 infective bites per person per year (PPY). During the last 2 years of follow-up (corresponding to the fourth and fifth years following the initiation of IRS: October 2017–October 2019), the daily HBR was 2.1 and the annual EIR was 0.43, corresponding to an over 15-fold decrease in anopheline biting and over 500-fold decrease in transmission intensity. Clinical indicators of malaria were considered only in children 0.5–10 years of age, as this age group was evaluated in both cohorts. Pre-IRS, the prevalence of microscopic parasitemia was 31.8%, the prevalence of microscopic or submicroscopic parasitemia was 67.5%, and the incidence of malaria was 2.96 episodes PPY. During the last 2 years of follow-up, the prevalence of microscopic parasitemia was 1.8%, the prevalence of microscopic or submicroscopic parasitemia was 6.8%, and the incidence of malaria was 0.05 episodes PPY, corresponding to a 10-fold reduction in the prevalence of any parasitemia and over 50-fold reduction in the incidence of malaria. Thus, great progress was made in decreasing the malaria burden, although a substantial reservoir of asymptomatic (largely submicroscopic) infections remained. These cohort studies also highlighted the effectiveness of providing prompt and effective antimalarial treatment. Over 3,000 episodes of malaria were diagnosed in both cohorts combined, but only four cases met criteria for severe malaria, and no malaria deaths occurred. Highly effective malaria control was also associated with other health benefits. Following the implementation of IRS mean hemoglobin levels increased significantly in children, with the overall prevalence of anemia reducing from to 3.4%.^11^ Over the 8-year observation period, care-seeking rates declined by 75% and the incidence of clinic visits at which antibiotics were prescribed to children declined by 70%.

In summary, our comprehensive cohort studies quantified dramatic reductions in malaria and nonmalaria indicators in an historically high-burden setting following the implementation of sustained malaria control interventions including access to prompt and effective antimalarial therapy, universal LLIN distribution, and IRS. However, despite these reductions, a substantial reservoir of asymptomatic infections remained, primarily among school-aged children and adults.^10^ In addition, nonadherence to LLINs (defined as participant not using a bed net the night prior to the survey as reported by the adult respondent during the household visits) after transmission declined was associated with an increased risk of malaria, highlighting the importance of LLIN use even in the setting of sustained IRS.^12^ Given the relatively high cost of IRS and the risk of resurgence following the withdrawal of IRS,^7^ further research is needed to facilitate effective IRS exit strategies. Furthermore, interventions in addition to those now widely available will likely be needed to achieve elimination in historically high-burden settings.

### Molecular epidemiology.

Conventional point-of-care malaria diagnostics, such as microscopy and rapid diagnostic tests (RDTs), do not have the sensitivity required to detect low-density infections, often referred to as submicroscopic or subpatent infections. More sensitive molecular techniques, such as LAMP (limit of detection 1–5 parasites/µL) or ultrasensitive *var*ATS qPCR (limit of detection 0.03–0.15 parasites/µL) can be used to detect these infections through active surveillance.^13^ Multiple studies have shown that submicroscopic infections are common across various transmission settings and are more frequent in adults than in children due to naturally acquired antimalarial immunity.^14^^,^^15^ Therefore, we have integrated these highly sensitive molecular assays as routine evaluations in our cohort studies to further characterize the burden and dynamics of asymptomatic infections.

In PRISM 1 prior to IRS, measured parasite prevalence was approximately twice as high in children and 8-fold higher in adults with results based on LAMP compared with microscopy alone,^16^ providing additional evidence that submicroscopic infections are common in high-transmission settings and that they become more frequent with increasing age. As transmission declined throughout the PRISM studies due to repeated rounds of IRS, the proportion of submicroscopic infections increased in all age groups; by the end of PRISM 2 in 2019, submicroscopic infections accounted for > 75% of infections in children (ages 0–15) and > 90% of infections in adults > 15 years of age^10^^,^^16^^,^^17^ These findings provided longitudinal data to support conclusions from previous cross-sectional studies that indicate an increase in the proportion of submicroscopic infections relative to microscopically detectable infections in settings, where malaria control efforts have been successful.^14^^,^^18^ Submicroscopic infections may also be clinically relevant; some studies suggest that low-density, chronic infections are associated with anemia, altered cognitive function, and/or systemic bacterial infection.^16^^,^^19^^,^^20^ In PRISM 1, children with submicroscopic parasitemia had an increased risk of fever and nonfebrile illness compared with children without parasitemia, suggesting that submicroscopic infections may have clinically relevant consequences for children.^21^

Our experience with the use of highly sensitive molecular diagnostics shows that these techniques are critical to fully characterize the parasite reservoir, given that microscopic infections represent only the “tip of the iceberg,” and that the relative proportion of submicroscopic infections increases with decreasing transmission intensity. Furthermore, these techniques can be used to identify asymptomatic individuals who provide persistent reservoirs of transmission in lower transmission settings, to identify infected populations who, although asymptomatic, may benefit clinically from treatment, and to evaluate the results of interventions.

**Figure 4.**
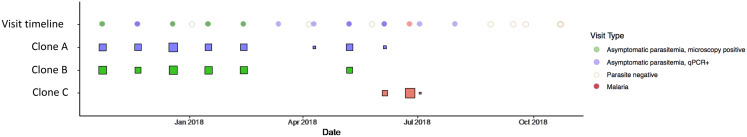
Top row shows the visit timeline of a participant along with parasitemia status. Apical membrane antigen-1 (AMA-1) genotyping reveals that this individual had a polyclonal infection with clones A and B that persisted until the participant was infected with a new clone that caused symptoms. After treatment of malaria, all parasite clones cleared. Given the oscillations in parasite density that occurred during the period of observation, which included some parasite negative visits, and the polyclonal nature of the infection, AMA-1 genotyping was essential to differentiate between chronic and new infections. This figure appears in color at www.ajtmh.org.

In addition to characterizing the parasite reservoir, highly sensitive molecular diagnostics can provide insight into longitudinal dynamics of infections. In PRISM 1 and 2, some individuals had low-density infections detected sequentially over numerous months. To further characterize the dynamics of these chronic infections, it is necessary to genotype parasite DNA to distinguish among individual clones (strains) as individuals in high-transmission settings are often coinfected with overlapping infections of multiple clones. Using genetic data, it is possible to follow distinct clones over time, which allows for assessment of the molecular force of infection (mFOI), or the incidence of genetically distinct parasite clones acquired over time, and estimation of the duration of infections.^22^ In the PRISM 2 cohort, we genotyped all samples with a parasite density ≥0.1 parasites/µL using amplicon deep-sequencing of the apical membrane antigen-1 (AMA-1) gene, a highly diverse region of the genome, allowing us to distinguish most parasite clones from each other (45 unique haplotypes detected).^23^^,^^24^ Infections with identical AMA-1 haplotypes were defined as genetically identical (from the same clone), which allowed differentiation of persistent from new infections (Figure 4). By using frequent sampling, ultrasensitive qPCR, and AMA-1 amplicon deep-sequencing, it was possible to accurately detect the onset of new infections and to estimate infection durations. This allowed us to estimate the clearance of asymptomatic *Plasmodium falciparum* infections and to investigate whether there were sex-based differences in clearance of these infections, given that we observed higher prevalence of asymptomatic infection in males compared with females. Analyzing the data by clone, where each unique clone was counted as an infection and each clone’s disappearance as a clearance event, we found that females cleared their asymptomatic infections nearly twice as quickly as males, even after adjusting for age, baseline infection status, and parasite density (adjusted hazard ratio for clearance of infection without antimalarial use = 1.82, 95% CI 1.20–2.75).^23^ There was no evidence for a sex-based difference in exposure to infection through behaviors or as measured by mFOI. Although previous studies have reported a higher prevalence of malaria infection in males compared with females, these differences were often ascribed to differences in exposure. However, in PRISM 2, the observed male bias in parasite prevalence was best explained by slower clearance of infections in males.

As described previously, amplicon deep-sequencing provides information vital to understanding the host response to malaria infection and better characterizes the dynamics of polyclonal asymptomatic infections compared with molecular methods that cannot differentiate among parasite clones. Because amplicon sequencing data can be used to estimate the mFOI, it can also be used to better understand changes in transmission in the setting of malaria control interventions. We plan to use amplicon deep-sequencing to assess changes in the mFOI and clinical antimalarial immunity in the setting of repeated rounds of IRS during longitudinal evaluation of the PRISM 1 and 2 cohorts.

### Assessing the human infectious reservoir for malaria.

Understanding who in the human population sustains transmission to mosquitoes is of great relevance for malaria control and elimination efforts.^25^ The increased use of molecular malaria diagnostics has established that submicroscopic infections are common in nearly all malaria endemic settings^15^ and that they may produce gametocytes at densities sufficient for transmission to mosquitoes.^26^ To examine infectivity to mosquitoes, an insectary with functioning mosquito membrane feeding facilities was established at our study site in Nagongera subcounty, Tororo District.^27^ In the PRISM 2 cohort, running from October 2017 to October 2019, gametocyte density and sex ratio were examined by molecular markers with mosquito feeding assays using a colony of *An. gambiae *s.s. mosquitoes.^28^ Key goals in these assessments were to determine: 1) the contribution to transmission of infections in relation to their detectability by different diagnostics (microscopy, molecular qPCR); 2) the contribution of different age groups to transmission; and 3) the duration of parasite carriage in relation to onward transmission potential.^17^

**Figure 5.**
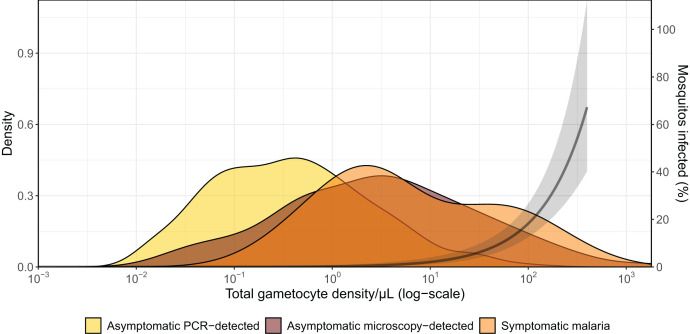
Gametocyte density distributions stratified by cohort participant infection and disease status. Relationship between gametocyte density and proportion of mosquitoes infected from membrane feeding assays. This figure appears in color at www.ajtmh.org.

Gametocyte prevalence among symptomatic malaria infections in the PRISM 2 cohort was 28.9%, considerably lower than that among asymptomatically infected individuals (67.6%; *P* = 0.033); this result suggests that in a cohort that is treated promptly whenever symptomatic malaria is diagnosed, most transmission will be from asymptomatic individuals. Mosquito feeding assays were successfully performed on both symptomatic and asymptomatic parasite carriers. At least one infected mosquito was observed in 7.2% of feeding experiments, with 1.2% of all examined mosquitoes becoming infected. The likelihood that a mosquito became infected rose rapidly when gametocyte densities exceeded 10 gametocytes/µL (assessed by molecular methods), corroborating findings from other African settings (Figure 5).^29^

Gametocyte density distributions differed among populations, and densities were highest overall in asymptomatic microscopy-detected malaria infections, and lower in clinical malaria cases and asymptomatic submicroscopic infections (Figure 5). After adjusting for gametocyte density, mosquito infection rates were lower for symptomatic malaria cases, suggesting that in symptomatic infections, gametocytes may be less mature or serum factors associated with inflammation may reduce gametocyte viability.^30^ When concurrently considering their prevalence in the population and their infectivity to mosquitoes, asymptomatic microscopy-positive individuals comprised 83.8% of the infectious reservoir, with asymptomatic submicroscopic infections responsible for 15.6% and symptomatic infections for only 0.6% of transmission. These results are broadly in line with direct assessments of the human infectious reservoir in Ethiopia^31^ and an indirect assessment from Western Kenya based on household-caught mosquitoes.^32^ In our study setting, children aged 5–15 years were responsible for 58.7% of the infected mosquitoes; individuals 16 years or older contributed the least to transmission among all age groups examined (15.6%). These population estimates may help in targeting malaria interventions to those demographics most important for malaria transmission,^33^^,^^34^ but they do not do justice to considerable heterogeneity in the infectivity of individual gametocyte carriers. Remarkably, four children (0.8% of the total population) were responsible for 62.6% of all infected mosquitoes. Some of the high-transmission infections in these children were chronic but others of short infection duration.

In summary, our findings suggest that passive detection of symptomatic infections or community mass screening and treatment based on conventional diagnostics might be insufficient to reduce the infectious reservoir and prevent onward transmission in settings of declining transmission.

### Antimalarial drug efficacy and resistance.

The treatment of malaria is challenged by resistance of *P. falciparum* to multiple drugs. Resistance to the older agents chloroquine and sulfadoxine-pyrimethamine (SP) has been common in Uganda for many years, and first-line therapy changed to the ACT AL in 2004. Sulfadoxine-pyrimethamine remains the standard-of-care for intermittent preventive treatment of malaria in pregnancy.

We have systematically studied antimalarial drug efficacy and resistance in Uganda for the last two decades, with therapeutic efficacy studies, characterization of genetic markers of resistance, and measurement of ex vivo drug susceptibilities of cultured isolates. Our results have demonstrated changing susceptibilities to key treatment regimens over time.

Evaluation of the therapeutic efficacy of chloroquine in 1998–1999 suggested poor efficacy,^35^^,^^36^ although early studies lacked genotyping to definitively assign outcomes. Subsequent trials demonstrated improved efficacy of SP, compared with chloroquine,^36^ and good efficacy for amodiaquine plus SP;^37^^–^^41^ although this combination was never widely established as a standard treatment, it is now widely used as seasonal malaria chemoprevention in parts of West and Central Africa.^42^

Trials of ACTs showed excellent antimalarial efficacies for artesunate-SP,^38^^,^^43^ artesunate-amodiaquine,^41^^,^^44^^–^^47^ AL,^44^^,^^45^^,^^47^^–^^51^ and dihydroartemisinin-piperaquine.^48^^–^^51^ After treatment with all tested ACTs, recrudescences were very uncommon, but new infections were common in areas of high-transmission intensity. Compared with other ACTs, treatment with dihydroartemisinin-piperaquine was followed by fewer recurrent infections, consistent with the long half-life of piperaquine,^49^ and the combination has been shown to be highly efficacious for chemoprevention in Ugandan children^52^^,^^53^ and pregnant women.^54^^,^^55^

**Figure 6.**
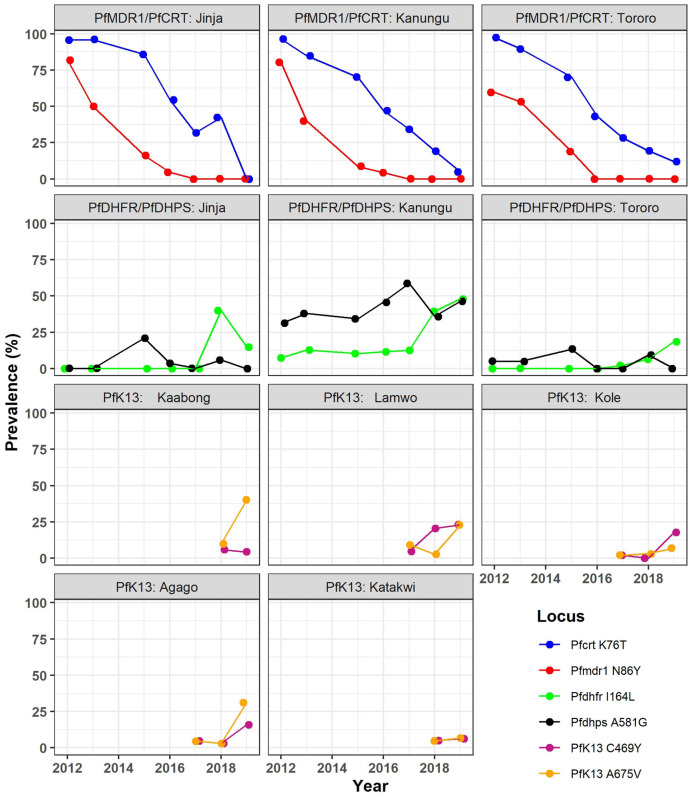
Prevalence of key drug resistance associated genetic polymorphisms at health centers in selected districts in Uganda. Prevalences are shown for the indicated years and districts. Jinja, Kanungu, and Tororo are in Central, Southwestern, and Eastern Uganda, respectively. The five districts for which PfK13 data are shown are all in Northern Uganda. This figure appears in color at www.ajtmh.org.

Considering molecular markers of resistance (Figure 6), serial studies in Tororo, in Eastern Uganda, showed steady declines over the last two decades in the prevalences of the aminoquinoline resistance markers *pfcrt* 76T and *pfmdr1* 86Y.^56^^–^^60^ Surveillance at sites across Uganda showed similar prevalences.^56^^,^^57^^,^^60^^,^^61^ Changes in resistance mediators over time are likely due to selective pressures of antimalarials, with evidence for opposite pressures on aminoquinoline resistance markers of regimens containing amodiaquine^62^^,^^63^ or piperaquine^64^ compared with lumefantrine.^65^ More recently described polymorphisms associated with resistance to piperaquine (other mutations in *pfcrt* and plasmepsin gene amplification) or lumefantrine (*pfmdr1* amplification) have shown very low prevalence in Uganda.^66^^,^^67^ Overall, the large majority of *P. falciparum* parasites now circulating in Uganda appear to be sensitive to available aminoquinolines and lumefantrine.

Considering SP, five mutations that mediate increasing levels of resistance showed > 90% prevalence in parasites collected across Uganda as early as 2002.^60^^,^^61^ Prevalences of two additional mutations that mediate high-level resistance to the components of SP, *pfdhfr* 164L, and *pfdhps* 581G, were very low through ∼2012, but have since increased, especially in Western Uganda, with prevalences near or exceeding 40% at multiple sites in 2018–2019.^56^^,^^57^ Thus, the antimalarial activity of SP, which has been compromised for many years, is now likely diminished even further.

Of recent concern is potential emergence of resistance to ACTs in Africa.^68^ About 20 PfK13 propeller domain mutations are validated or candidate markers of delayed parasite clearance after treatment with artemisinins, generally referred to as artemisinin resistance.^69^ Consistent with studies across Africa,^70^ older studies from Uganda showed very low prevalence of validated/candidate PfK13 mutations.^60^^,^^71^^–^^73^ In contrast, recent surveillance has shown emergence of parasites with either of two PfK13 candidate resistance mutations, 469Y and 675V,^57^^,^^74^ with prevalences increasing to 20–30% in multiple districts of Northern Uganda in 2018–2019.^56^ Another validated PfK13 mutation, 561H, was seen at similar prevalences in Central Rwanda.^75^^–^^77^ Recent studies have shown the Ugandan^78^ and Rwandan^77^ mutations to be associated with delayed parasite clearance after treatment with artemisinins. Thus, evidence is now strong that artemisinin resistance has emerged in East Africa, and specifically in Northern Uganda. However, drug efficacy studies have not recently been performed in relevant regions of Uganda, and in Rwanda, PfK13 561H was not clearly associated with AL treatment failure. Thus, although emergence of resistance-mediating PfK13 mutations is very concerning, impacts on antimalarial treatment efficacy remain uncertain.

Ex vivo analyses of cultured parasites have shown results consistent with molecular studies of resistance markers. Isolates collected in Kampala in 2006–2008 demonstrated chloroquine susceptibilities suggesting high-level resistance.^79^ More recently, with the reversion to wild type sequences described above, > 90% of isolates collected in Eastern Uganda had IC_50_s < 100 nM, consistent with chloroquine sensitivity, although a minority had high IC_50_s.^66^^,^^67^^,^^80^ Susceptibilities to the key ACT partner drugs lumefantrine, amodiaquine, piperaquine, mefloquine, and pyronaridine were all generally excellent.^66^^,^^67^^,^^80^ In older studies, the ring survival assay (RSA), a laboratory marker for artemisinin resistance, showed sensitive isolates in Kampala^72^ and Tororo.^66^^,^^67^ However, parasites from Northern Uganda with PfK13 675V were recently shown to have abnormal RSAs,^78^ and additional genotype–phenotype association studies are underway.

In summary, surveillance over the last two decades has demonstrated remarkable changes in drug resistance profiles of Ugandan malaria parasites. Recent results suggest a return to chloroquine sensitivity for most, but not all circulating parasites; major limitations in the antimalarial chemopreventive activity of SP; and worrisome emergence of artemisinin resistance in Northern Uganda. There is an urgent need for continued surveillance and clinical trials to determine whether current regimens for the treatment and prevention of malaria in Uganda will need to be replaced.

### Insecticide resistance.

The dramatic scale-up of malaria vector control interventions across Africa, including LLINs and IRS, has been associated with an estimated 40% decrease in the incidence of disease between 2000 and 2015.^81^ In tandem with global gains, expanded coverage of LLINs and IRS in Uganda has been associated with declines in the prevalence of malaria in children under 5, from 19% in 2014 to 9% in 2018.^82^ Although a variety of insecticides are deployed through IRS, all widely available LLINs are treated with pyrethroids, and reports of increasing resistance to this class of insecticides are highly concerning.^83^ Pyrethroid resistance is commonly mediated through two main mechanisms including “knock down resistance” caused by target-site mutations in the receptor for pyrethroids (kdr),^84^^,^^85^ and metabolic resistance mediated by cytochrome p450 enzymes.^86^ To combat pyrethroid resistance, dual active ingredient LLINs have been developed, which combine pyrethroid insecticides with other agents, including PBO, a synergist;^87^ chlorfenapyr, a proinsecticide;^88^ and pyriproxyfen, an insect growth regulator.^89^

In our PRISM projects, we have conducted entomologic surveillance, monitored for insecticide resistance, and assessed the impact of vector control interventions on *Anopheles* mosquitoes.^90^ In Nagongera, Tororo district, near collapse of *An. gambiae *s.s. and *An. funestus* populations was observed following universal distribution of LLINs plus multiple rounds of IRS, with the more behaviorally resilient *An. arabiensis* becoming the predominant vector species.^90^ In Tororo, these interventions were also associated with declines in the indoor HBR and sporozoite infections, and increased outdoor biting.^91^ Routine monitoring of insecticide resistance to pyrethroids revealed high levels of resistance to both class I (permethrin) and class II (deltamethrin) pyrethroids,^83^ and to carbamates (bendiocarb).^92^ However, no resistance to the organophosphate pirimiphos-methyl, which is routinely used for IRS by the NMCD, was observed. We recorded evidence of contemporary gene flow between sympatric *An. gambiae s.s* and *An. arabiensis*,^93^ which is of particular concern, as it may result in the transfer of adaptively important genetic traits such as insecticide resistance between these two species.

Through our PRISM projects, we have established foundations and collaborations that have been leveraged to conduct additional research on insecticide resistance,^94^ and contributed to mapping of insecticide resistance in Africa.^95^ Improved molecular analysis tools and increased surveillance for genetic markers of insecticide resistance has been propelled by availability of the *An. gambiae* genome;^96^ PRISM contributed > 100 samples toward its development.^96^ The *An. gambiae* genome has enabled exploration of gene targets with putative association with insecticide resistance. The PRISM projects used this platform to aid the discovery of two novel variants (*Cyp4j5* and *Coeaeld*),^97^ which are associated with resistance to pyrethroids. These markers appear to be widely spread in Uganda, approaching fixation in some areas.^94^ Overall, our insecticide resistance monitoring has demonstrated high levels of resistance to pyrethroids, appreciable impact of the synergist PBO to pyrethroid susceptibility, emerging resistance to carbamates, and complete susceptibility of malaria vectors to organophosphates, underscoring the importance of this class to current insecticide resistance management strategies.

## CONCLUSION

Utilizing comprehensive malaria surveillance studies, we demonstrated reductions in measures of transmission, infection, and disease following roll out of effective malaria control interventions. Similar trends were seen and conclusions drawn from data collected from both enhanced health facility-based surveillance and comprehensive cohort studies, highlighting our understanding that both surveillance methods are effective tools for quantifying the impacts of population-level malaria control inventions. We also demonstrated that although younger children are at greatest risk of malaria, school-aged children are the main reservoir of infection and drivers of transmission. This finding is particularly true in settings, where recent malaria control efforts have been successful. Thus, with the goal of reducing human to mosquito transmission, school-aged children may be an ideal group to target for control interventions. We have shown that surveillance of drug and insecticide resistance is critical for informing policy, as evidenced by recent emergence of markers predicting resistance to artemisinin antimalarials and expanding vector resistance to multiple classes of commonly used insecticides. With a recent emphasis on the high burden to high impact approach in countries such as Uganda, our ICEMR program is committed to providing valuable data to support an evidence-based approach to reducing the burden of malaria, eventually moving toward elimination.
